# Limited relationships between reactive oxygen species levels in culture media and zygote and embryo development

**DOI:** 10.1007/s10815-018-1363-6

**Published:** 2018-11-10

**Authors:** Kuo-Chung Lan, Yi-Chi Lin, Yung-Chiao Chang, Hsin-Jung Lin, Yi-Ru Tsai, Hong-Yo Kang

**Affiliations:** 1 Department of Obstetrics and Gynecology, Kaohsiung Chang Gung Memorial Hospital and Chang Gung University College of Medicine, 123 Ta-Pei Road, Niao-Sung District, Kaohsiung city, Taiwan; 2 Center for Menopause and Reproductive Medicine Research, Kaohsiung Chang Gung Memorial Hospital and Chang Gung University College of Medicine, Kaohsiung, Taiwan; 3 Department of Obstetrics and Gynecology, Chiayi Chang Gung Memorial Hospital, Chiayi, Taiwan

**Keywords:** Culture media, Embryo morphology, Reactive oxygen species, Fertilization, Embryo development

## Abstract

**Purpose:**

Reactive oxygen species (ROS) are thought to play a critical role in the success of IVF. The relationships between oxidative stress parameters in culture media and IVF outcomes have not been extensively investigated. The objective of this study is to examine the relationships between early human embryonic parameters and levels of ROS in culture media.

**Methods:**

This prospective study was conducted with 2633 spent culture media collected from patients undergoing conventional IVF (*n* = 101) and intracytoplasmic sperm injection (ICSI) (*n* = 60). Both fertilization and early culture were performed in universal IVF medium and G series medium. ROS levels were measured by chemiluminescence assays using luminol as the probe on days 1, 3, and 5 and determined the relationships of ROS levels with zygote condition, embryo quality, and clinical pregnancy rate.

**Results:**

ROS levels per embryo in culture media on the corresponding days 1, 3, and 5 showed significant correlations between each pair in the total cohort. Similar results were observed in the IVF and ICSI groups, but day 1 and day 3 ROS levels were significantly higher in culture media of IVF than of ICSI embryos. ROS levels in culture medium were not significantly associated with embryo quality, blastocyst formation, or arrest. ROS levels on day 1 were similar in media of normally fertilized zygotes, unfertilized oocytes, and polyspermic zygotes and were not associated with delayed embryonic development, high fragmentation, blastocyst formation, or arrest after prolonged culture. ROS levels in media were not associated with the likelihood of conception.

**Conclusions:**

ROS levels in culture media may not be an effective indicator of embryo selection for IVF.

## Introduction

The need to identify the most viable embryo following in vitro fertilization (IVF) is crucial to enhance successful pregnancy rates. Identification and transfer of single embryos of known viability and high developmental potential can avoid multiple pregnancies [1]. The standard method of identifying the best embryos has been morphology [2]. Morphologic examination has increased knowledge of the optimal appearance of embryos at different developmental stages, with optimal appearance closely related to success. However, despite the accumulation of valuable morphological data and experience, all of these assessment methods rely on the same visual depiction of embryos.

Other proposed embryo selection techniques include preimplantation genetic screening, despite concerns over its invasive nature [3]. Alternative noninvasive methods to determine embryo viability have included measurements of factors in growth media or imaging of the embryo [4]. May in the future allow embryologists to distinguish between viable and non-viable oocytes and embryos.

Optimizing the composition of embryo culture media and incubation settings is essential for enhancing embryo quality during in vitro development. Reactive oxygen species (ROS) in the culture media may originate from metabolism by the embryo and/or its surroundings. ROS are thought to play a critical role in the success of IVF [5]. Oxidative stress has been defined as increases in the steady-state levels of various ROS, exceeding the body’s antioxidant defenses [6]. Oxidative stress can not only alter many types of cellular molecules, but can also block or retard early embryonic development [7].

Concentrations of ROS in spent culture media have been reported to correlate with an advanced degree of embryo fragmentation or blastocyst formation [8–11]. The exact role of ROS in early embryonic development has yet to be fully determined. In contrast, the use of commercial media in culture systems has improved the consistency of embryo culture. Culture media are frequently supplemented with antioxidants, thereby maintaining a pro-oxidant-antioxidant equilibrium in embryos [5]. Embryos in commercial culture media have been found to generate ROS at various rates, depending on the composition of the media [10, 12]. Nevertheless, the relationship between oxidative stress parameters in culture media and IVF outcome in qualified, stable embryological laboratories has not been extensively investigated. It remains unclear whether culture media can neutralize excess ROS levels, thereby protecting cultured embryos and preventing the detrimental effects of ROS. Furthermore, it has not been determined whether ROS measured in culture media can provide information about embryo quality not provided by morphology. The present study assessed ROS levels in culture media, embryos were cultured individually, of women undergoing IVF/intracytoplasmic sperm injection (ICSI) and determined the relationships of ROS levels with embryo development and quality.

## Materials and methods

### Patients

This study was approved by the Institutional Review Board (IRB) of Chang Gung Memorial Hospital (CGMH98-4000B). A total of 161 women undergoing first successfully oocyte retrieval cycles of assisted reproduction, including 101 IVF and 60 ICSI cycles, between December 1, 2010, and December 1, 2012, were prospectively included. Two thousand six hundred thirty-three spent culture media were collected. All couples completed the standard infertility workup; couples were not selected for age, sperm parameters, or causes of infertility.

### Controlled ovarian hyperstimulation

The protocol for controlled ovarian hyperstimulation followed the standard downregulation regimen of our institution [13, 14]. All women were administered once daily subcutaneous injections of leuprolide acetate (LA) (Lupron, Abbott, North Chicago, IL, USA), at doses of 1 mg for those aged < 35 years and 0.5 mg for those aged ≥ 35 years, beginning on day 21 of the previous cycle and lasting until cycle day 3. Pituitary downregulation was evaluated by measuring serum estradiol (E2) concentration using a commercially available competitive immunoassay with the IMMULITE Analyzer (DPC Coat-a Count; Diagnostic Products Corp., Los Angeles, CA, USA) and by transvaginal sonography (TVS) of the ovaries. If serum E2 level was < 35 pg/mL and TVS detected no follicles > 10 mm in diameter, LA doses were reduced 50% and continued up until and including the day of human chorionic gonadotrophin (hCG) administration.

If the pituitary was not suppressed, LA was continued at the full dose and serum E2 concentration was rechecked every 3 days until suppression was achieved. Following pituitary suppression, patients aged < 35 and ≥ 35 years were administered 225 IU/day and 300 IU/day rFSH (Gonal-F, 75 IU/ampoule, Serono, Aubonne, Switzerland), respectively, for 5 days, after which the dose was individualized based on ovarian follicular growth. Patients were monitored by TVS and serum E2 concentrations were measured every 2 to 3 days, starting on the fifth day of stimulation. Patients were administered hCG (Ovidrel®; Serono, Modugno, Italy) when at least two follicles were ≥ 18 mm in diameter and serum E2 levels were adequate. Progesterone was measured only on the day of hCG administration. Oocytes were retrieved 34–36 h after hCG administration.

### Oocyte and sperm preparation

For IVF, the retrieved oocyte-corona-cumulus complexes were immediately classified according to their maturity and cultured in universal IVF medium (Medicult, Copenhagen, Denmark) for 4–6 h before insemination. For ICSI, the oocyte-corona-cumulus complexes were denuded and assessed 2–4 h after retrieval. These complexes were placed in medium containing 80 IU/mL of hyaluronidase for 5 s. The cumulus and corona of the cells were removed mechanically using a set of pipettes with consecutive inner diameters of 220, 180, and 140 μm. The nuclear maturation grades of the oocytes were classified as metaphase II (MII) or non-MII, with the latter including metaphase I and prophase I (PI) oocytes. The denuded oocytes were cultured in universal IVF medium and examined for the presence of the first polar body, with ICSI performed after the first polar body was confirmed. The oocytes that did not develop to MII after 8 h of incubation were discarded.

After 2 days of abstinence, semen samples were collected from the male partner by masturbation into a sterile container. Motile spermatozoa were selected using a discontinuous two-layer density gradient technique (SupraSperm 80/55; Origio, Malov, Denmark). The retrieved oocytes were inseminated 4–6 h later with 50,000 spermatozoa in NUNC 4 well culture dishes, with each well containing 700 μL universal IVF medium. After 16–18 h, the cumulus cells were removed with pipettes of internal diameter 170 μm and fertilization was checked. For ICSI procedures, cumulus cells were removed 2 h after oocyte retrieval using 80 IU/mL hyaluronidase (H-3757; Sigma Chemical, St. Louis, MO, USA) in HTF. ICSI was performed 2–4 h after denuding.

### Assessment of fertilization, embryo culture, and zygote and embryo grading

Fertilization was confirmed 16 to 18 h subsequent to IVF or ICSI. A total of 882 two-pronuclei zygotes, 40 polyspermic zygotes, and 111 unfertilized oocytes from the 161 cycles were investigated. Day 1 zygotes were scored as described [13, 14], based on the size and alignment of nuclei and the number and distribution of nucleoli. Briefly, zygotes with equal numbers of nucleoli aligned at the pronuclear junction were classified as Z-1; absolute numbers were not counted but were between three and seven. Zygotes with equal numbers and sizes of nucleoli (between three and seven) equally distributed in the two nuclei were classified as Z-2. Zygotes with equal numbers of nucleoli of equal sizes in the same nuclei, but with one nucleus showing nucleolar alignment at the pronuclear junction and the other having scattered nucleoli, were classified as Z-3. Zygotes with unequal numbers (differences > 1) and/or sizes of nucleoli were also classified as Z-3. Zygotes with pronuclei that were not aligned, were of grossly different sizes, or were not located in the central part were classified as Z-4. Embryos were cultured in G1™ v5 medium (Vitrolife Sweden AB, Vastra Frolunda, Sweden) on days 1–3 and in G2™ v5 medium (Vitrolife Sweden AB) on days 3–5 or 6. Day 3 embryos were evaluated (66–68 h postinsemination/ICSI, respectively) and were classified using a modification of Veeck’s morphologic grading system [15], with grade I defined as 7, 8, and 9 cells, with blastomeres of equal size and no cytoplasmic fragments; grade II as 7, 8, and 9 cells, with blastomeres of equal size and < 20% cytoplasmic fragments; grade III as 7, 8, and 9 cells, with uneven blastomere sizes and no cytoplasmic fragments; and grade IV as embryos with > 20% fragmentation or pre-embryos with few blastomeres of any size and with major or complete fragmentation. After 2 days of culture in G2™ v5 medium, blastocyst formation was evaluated (114–116 h postinsemination/ICSI, respectively) in day 5 embryos, with scoring based on the expansion state of the blastocyst and on the consistency of the inner cell mass and trophectoderm cells. Class 1 consisted of full blastocysts onward; an inner cell mass with many tightly packed cells; and a trophectoderm with many cells forming a cohesive epithelium; class 2 as an inner cell mass with loosely packed cells and/or a trophectoderm with loose cells forming a cohesive epithelium, or a day 5 morula with compaction; class 3 as an inner cell mass with few loosely packed cells and/or a trophectoderm with loose cells forming a cohesive epithelium, or a day 5 morula without compaction; and class 4 as arrested embryos. Embryo survival was assessed based on morphology and cleavage speed. Embryos with the same number of blastomeres at two consecutive observations, and zygotes that remained blocked at the pronuclear stage, were considered to be developmentally arrested. A single team of embryologists coordinated all procedures, thereby ensuring that both the culture protocols and the embryo assessment were standardized. The quality control team used a certified thermometer with a specially designed microprobe to ensure the droplet temperature remained at 37 ± 0.1 °C inside each chamber of the incubator (Scientific HERACELL 150i) with internal HEPA filters as well as a gas supply that is filtered through Coda® Inline filter cartridges (LifeGlobal). They also maintained the O_2_ level inside the incubator at 5% and the cultivating medium pH at 7.35 ± 0.01 with CO_2_ around 5.3%. During the study, all disposables, media, and oil underwent a one-cell mouse embryo assay and sperm bioassay to exclude embryo toxicity.

### ROS measurement

The corresponding zygotes (day 1) and embryo development (day 3 or day 5) were recorded and the spent culture media were also collected for determination of ROS levels. After evaluating the oocytes for fertilization 16–18 h after insemination or ICSI, the insemination media in the central wells of the culture dish were collected, as were the control media in the outer wells of the culture dish undergoing the same culture process. Both were immediately prepared for ROS measurement. After evaluating the embryos the morning of day 3 or day 5, the culture media were also collected as the test sample or as the control.

ROS levels in culture media were evaluated by a chemiluminescence assay using luminol (5-amino-2,3-dihydro-1,4-phthalazinedione) as a probe. Briefly, 10 μL luminol (1 M) in DMSO was added to 100 μL of culture medium in the OptiPlate-96 microplates (PerkinElmer). Chemiluminescence was evaluated with a luminometer (Wallac, Victor2 V, PerkinElmer) in the integration mode at 37 °C for 10 min after luminol was added (the media in the outer wells of the culture dish as negative control; 3% H_2_O_2_ as positive control). ROS levels were expressed as counts per second (cps).

### Establishment and follow-up of pregnancy

Embryos were replaced transcervically into the uterus on day 3 or 5 after oocyte retrieval, depending on each individual. Our center routinely offers blastocyst transfer (BT) to patients with ≥ 3 eight-cell embryos on day 3. Patients with < 3 eight-cell embryos on day 3 underwent day 3 embryo transfer.

Beginning on the day after oocyte retrieval, women received luteal phase supplementation, by once daily intravaginal administration of progesterone (90 mg) vaginal gel (Crinone 8%, Serono Pharmaceuticals Ltd.) or 800 mg micronized progesterone (Utrogestan, Piette International Laboratories, Belgium). Pregnancy was confirmed by detecting hCG in the urine 2 weeks after transfer. Clinical pregnancy was defined as the detection of a gestational sac at 7 weeks by transvaginal ultrasonography. If conception had occurred, progesterone supplementation was provided for an additional 4 weeks. The clinical pregnancy rate was calculated by dividing the number of cycles with fetal cardiac activity on ultrasound by the number of embryo transfer cycles, and the implantation rate was calculated by dividing the number of gestational sacs on ultrasound by the number of embryos transferred.

### Statistical analysis

ROS levels, PRs, and other parameters were analyzed in the entire cohort and separately in the IVF and ICSI groups. All statistical analyses were performed with SPSS 23.0 software (Statistical Package for Social Sciences, Inc., Chicago, IL, USA). Descriptive statistics are presented as frequency (percentage) or mean. Continuous data were summarized as the mean ± standard deviation. The variables were investigated using the Kolmogorov-Smirnov test to determine whether or not they are normally distributed. When the variables were normal distributed, two independent *t* tests were used to compare. When the variables were not normal distributed, means were compared using the Mann-Whitney rank-sum test. The chi-square and Fisher’s exact tests, where appropriate, were used to compare the proportions of the groups. Associations with quantitative and ordinal variables were assessed using a logistic or linear regression model. All *P* values were two-sided, with *P* < 0.05 considered statistically significant.

## Results

### Demographic variables and assessment of cycle outcome (Table 1)

The demographic features of the study population and the indications for assisted reproduction are presented in Table 1. A total of 161 patients underwent first successfully oocyte retrieval cycles of assisted reproduction, including 101 IVF cycles and 60 ICSI cycles, during the study period. One thousand thirty-three day 1, 963 day 3, and 637 day 5 spent culture media were collected. Mean ROS levels in culture media on cycle days 1, 3, and 5 are also shown in Table 1.

**Table 1:** Demographic characteristics of the 161 IVF/ICSI cycles

No. of cycles with at least 1 oocyte retrieved	161
No. of cycles that underwent IVF	101
No. of cycles that underwent ICSI	60
Age of female partner (years)	34.9 ± 4.7 (21–45)
Body mass index (kg/m^2^)	21.7 ± 2.8 (15.6–33.3)
Infertility	
Primary	121
Secondary	40
Duration of infertility (years)	4.3 ± 3.0 (1–16)
No. of mature or nearly mature oocytes retrieved	6.6 ± 3.6 (1–21)
Endometrial thickness on day of hCG (mm)	1.3 ± 0.2
Estradiol (pg/mL) on hCG day	2688.9 ± 1803.6
Progesterone (pg/mL) on hCG day	1.1 ± 0.7
No. of indications	
Tubal factor	31
Male	45
Endometriosis	5
Ovulatory factor	32
Unexplained and others	10
Combined factors	38
ROS level (cps)	
Day 1	65.4 ± 47.4
Day 3	155.1 ± 116.7
Day 5	63.1 ± 29.2
Normal fertilization rate	85.3% (882/1033)
No. of embryos transferred	2.3 ± 1.0 (1–4)
No. of cycles with freezing all embryos or no embryos available	6
Clinical pregnancy rate/transfer cycle	39.4% (61/155)
Implantation rate	24.8% (96/387)

Values are mean ± SD or proportion

### Relationships of day 1, 3, and 5 ROS levels in culture media

**Fig. 1:**
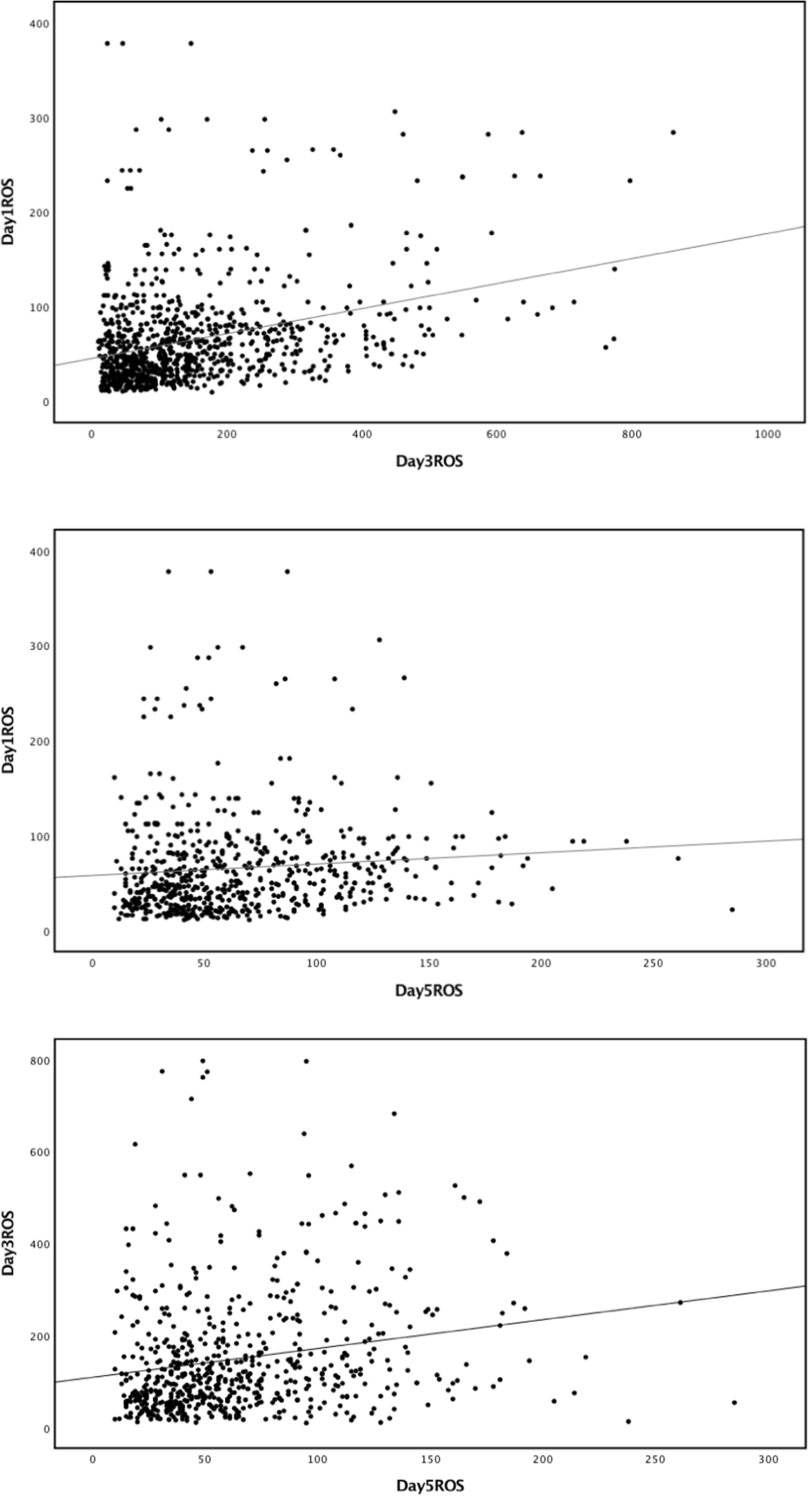
Correlations between ROS levels in spent culture media on days 1 (*n* = 1033), 3 (*n* = 963), and 5 (*n* = 637). **a** Day 1 vs. day 3 (regression coefficient: 0.336, *P* < 0.001). **b** Day 1 vs. day 5 (regression coefficient: 0.093, *P* = 0.02). **c** Day 3 vs. day 5 (regression coefficient: 0.196, *P* < 0.001). Straight lines represent the linear regression lines. ROS reactive oxygen species, *n* number

Linear regression analysis of the correlations of ROS levels per embryo in culture media on days 1, 3, and 5 showed significant correlations between each pair in the total cohort (Fig. 1) and separately in the IVF and ICSI groups. Regression coefficients on days 1 and 3, days 1 and 5, and days 3 and 5 were 0.468, 0.151, and 0.164, respectively, in the ICSI group and 0.312, 0.101, and 0.212, respectively, in the IVF group.

### Comparison of ROS levels per embryo in IVF and ICSI cycles

Patient age did not differ significantly in the IVF and ICSI groups. However, ROS levels in culture media on days 1 and 3, but not on day 5, were significantly higher for IVF than for ICSI embryos (Table 2).

**Table 2:** ROS levels in the culture media during IVF (*n* = 101) and ICSI (*n* = 60) cycles

Variable	IVF cycles (*n* = 101)	ICSI cycles (*n* = 60)	*P* value
Age (years)	34.2 ± 4.1	35.5 ± 5.2	NS
Day 1 ROS (cps) (sample)	69.9 ± 56.8 (*n* = 727)	51.2 ± 34.4 (*n* = 305)	< 0.001
Day 3 ROS (cps) (sample)	158.2 ± 142.2 (*n* = 687)	126.6 ± 117.3 (*n* = 297)	0.001
Day 5 ROS (cps) (sample)	64.1 ± 40.3 (*n* = 458)	68.9 ± 44.5 (*n* = 197)	NS

All values reported as mean ± SD

*n* number, *NS* not significant

### Relationship of ROS in culture media with zygote and embryo development

**Fig. 2:**
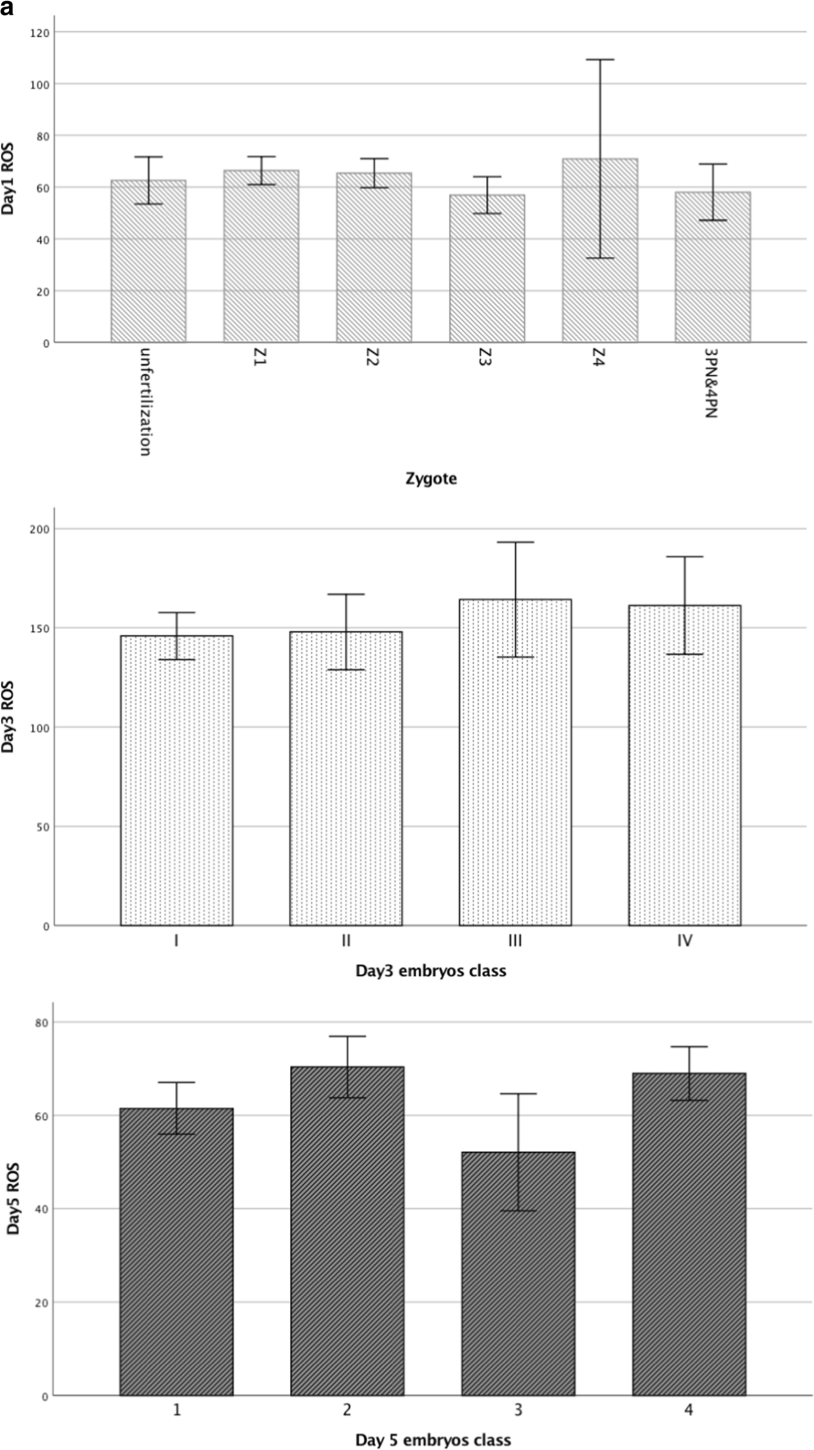

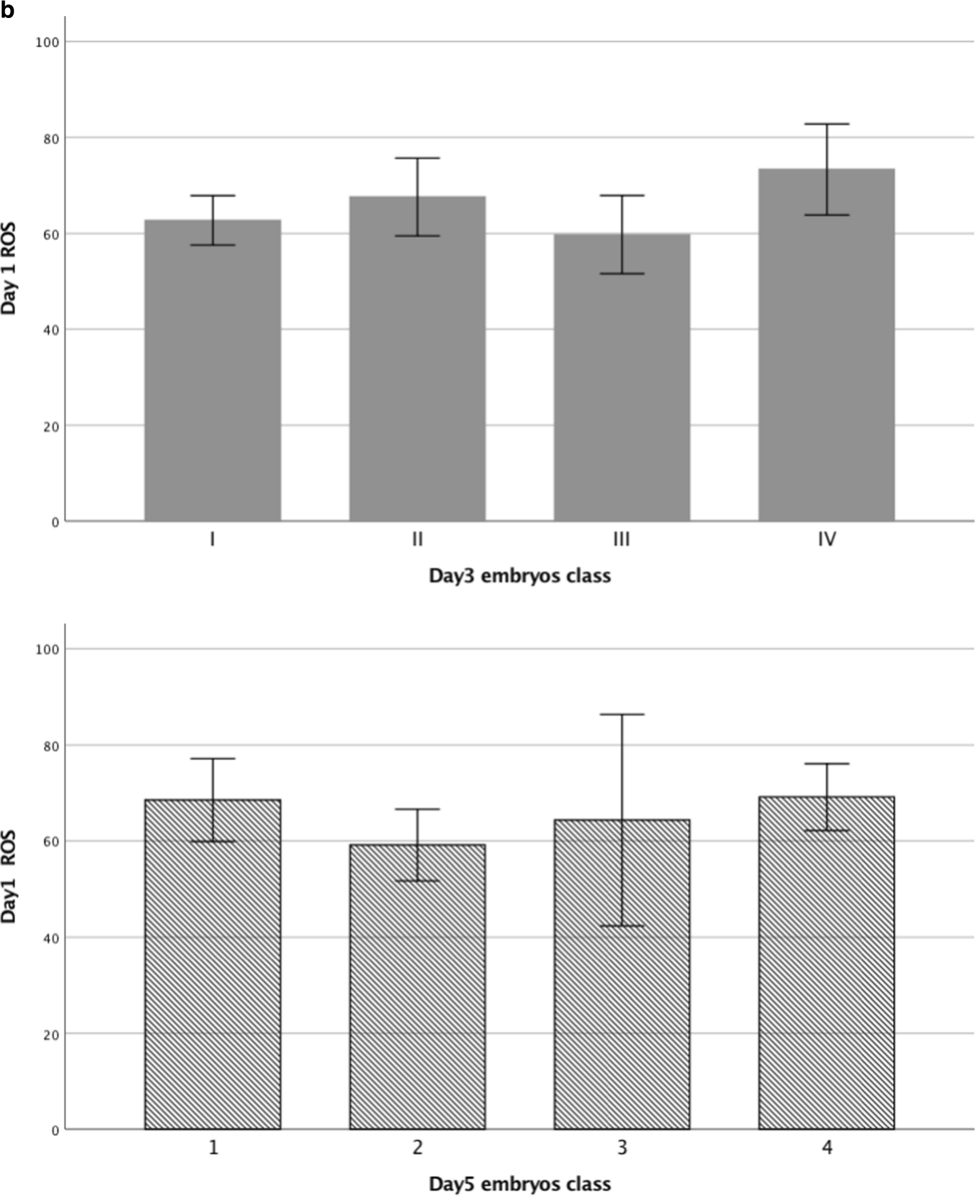
Relationship of ROS in culture media with zygote and embryo development. **a** Relationships between ROS levels in culture media on days 1, 3, and 5 with zygote quality, day 3 embryo class, and day 5 blastocyst class. **b** Relationship between ROS levels in culture media on day 1 with day 3 embryo class and day 5 blastocyst class

ROS levels in culture media on days 1, 3, and 5 were unrelated to indicators of corresponding embryo development, including zygotes, embryo quality, embryo arrest or survival, or blastocyst grading (Fig. 2a). ROS levels on day 1 were similar in media of normally fertilized zygotes with different scores, unfertilized oocytes, and abnormal polyspermic zygotes (Fig. 2a). Furthermore, ROS levels on day 1 media were also not associated with corresponding day 3 embryos or day 5 blastocyst class (Fig. 2b). Similar findings were observed when the IVF and ICSI groups were analyzed separately.

### Comparison of demographic variables in pregnant and non-pregnant patients

Of the 155 fresh transfer cycles analyzed, 61 resulted in pregnancy and 94 did not. Variables associated with pregnancy, including maternal and treatment characteristics, were analyzed (Table 3). Significant differences in age, number of mature oocytes collected, progesterone concentration on hCG day, and the number and score of embryos transferred were observed between the pregnancy and non-pregnancy cycles. In contrast, there were no significant differences in body mass index, endometrium thickness, gonadotropin dose, days of gonadotropin stimulation, and ROS levels corresponding transferred embryos in culture media.

**Table 3:** Demographic, clinical, and laboratory factors associated with the occurrence of pregnancy

Variable	No pregnancy (*n* = 94)	Pregnancy (*n* = 61)	*P* value
Age (years)	35.6 ± 4.9	34.0 ± 4.2	0.038
BMI, kg/m^2^	21.6 ± 2.7	21.8 ± 3.0	NS
Ampoules of 75 IU FSH	29.9 ± 10.0	29.7 ± 12.1	NS
Injection days	8.9 ± 1.7	9.1 ± 1.5	NS
Endometrial thickness on day of hCG (mm)	1.3 ± 0.3	1.3 ± 0.3	NS
Mean no. of mature oocytes collected	5.8 ± 3.1	7.3 ± 3.8	0.012
Progesterone on hCG day (ng/mL)	1.2 ± 0.8	0.9 ± 0.4	0.006
Estradiol on hCG day (pg/mL)	2521.2 ± 1845.6	2851.0 ± 1711.7	NS
No. of embryos transferred	1.7 ± 1.1	2.5 ± 0.8	< 0.001
Scores of embryos transferred	5.4 ± 4.0	8.8 ± 3.1	< 0.001
Day 1 ROS levels of embryos transferred (cps)	68.1 ± 58.4	62.5 ± 41.0	NS
Day 3 ROS levels of embryos transferred (cps)	156.8 ± 125.3	136.1 ± 97.5	NS
Day 5 ROS levels of embryos transferred (cps)	64.2 ± 34.1	54.9 ± 26.4	NS

Results are expressed as mean ± SD

*NS* not significant

## Discussion

Although morphologic scoring is the best available method of embryo selection, measuring markers of oxidative stress, such as ROS levels, in culture media may be a non-invasive alternative or may be combined with conventional morphology score to provide more precise embryo selection. This study, however, demonstrated that ROS levels in culture media are not significantly associated with zygote score, embryo quality, blastocyst formation or arrest, or chances of conception. Our findings indicate that age and embryo quality are the major predictors of pregnancy outcomes in women undergoing IVF/ICSI [16]. ROS levels in culture media could not distinguish between conception and non-conception cycles.

Under physiological conditions, ROS and antioxidants maintain a stable ratio. Theoretically, excess oxidative stress can have deleterious effects on the cellular milieu, negatively affecting fertilization, impairing cellular growth in the embryo, or inducing apoptosis, resulting in embryo fragmentation [17].

Although we observed positive correlations among ROS levels on days 1, 3, and 5, ROS levels on day 1 had no effect on zygote grade, unfertilized oocytes, or abnormal polyspermic zygotes. Furthermore, ROS levels on day 3 were not significantly associated with cleavage stage embryo quality, and ROS levels in culture media on day 5 were unrelated to embryo arrest or blastocyst formation or grading.

In contrast to our findings, higher day 1 ROS levels in culture media were reported to be associated with delayed embryonic development, higher fragmentation rates, and enhanced rates of development of morphologically abnormal blastocysts after prolonged culture [11]. These findings suggested that ROS generated in culture media by day 3 may be an important biochemical marker for blastulation [9].

It is difficult to compare our results with those of previous studies, as embryos in our study were cultured individually. In other studies, however, groups of 4–5 embryos were cultured together, making it difficult to assess the ROS levels in the microenvironment of each embryo.

Factors added to culture media have been shown to improve the oxidative state of early embryos. Many of these factors, especially human serum albumin, scavenge ROS and confer protection against DNA damage [18]. The embryo has several mechanisms to defend against ROS [19]. ROS can be induced by several factors, including exposure to visible light, the composition of the culture media, temperature and pH, oxygen concentration, centrifugation during spermatozoa preparation, ART techniques involving gamete/embryo handling, and cryopreservation methods [5]. These factors increase oxidative stress and result in suboptimal ART outcomes. Admittedly, the utmost care is required to avoid inducing excess ROS, and our results suggest that assessment of oxidative stress parameters in culture media has little effect on ART outcomes.

Although physiological concentrations of ROS are necessary for normal reproductive function in vivo, in vitro manipulation of gametes and embryos may expose these cells to excess ROS, generated by endogenous or exogenous environmental factors. Endogenously, the gametes and the developing embryo become sources of ROS [11], and ROS can be generated by spermatozoa and leucocytes, as well as by events such as sperm-mediated oocyte activation and activation of the embryonic genome [20].

In contrast, we found that ROS levels in day 1 zygote culture media differed significantly in the IVF and ICSI groups, findings consistent with previous results [6, 8]. The potential cellular sources of ROS differ in conventional IVF and ICSI. In conventional IVF, ROS in culture media may originate from the oocytes, the cumulus cell mass, and the spermatozoa used for insemination, with intracellular mechanisms in sperm generating ROS at the level of the plasma membrane and mitochondria [5].

On the other hand, the defective remaining sperm that have limited antioxidant defenses that surround the oocyte may also affect the production of ROS and other toxic factors into the IVF culture media during in vitro incubation [5]. It has been suggested the accumulation of these toxic factors could be avoided by either performing intracytoplasmic sperm injection (ICSI) or by shortening the time of sperm contact with the oocyte during incubation in IVF [5].

In ICSI, cumulus cells are not potential sources of ROS because incubation is initiated after denuding the oocytes from cumulus cells. The potential cellular sources of ROS in ICSI include spermatozoa and injected oocytes. The contact time between sperm and oocytes is also minimized, reducing damage induced by ROS-producing defective spermatozoa. Although the ICSI procedure might induce stress or shearing force on the plasma membrane of the oocyte, the deleterious effects of oxidative stress are thought to be due to a lack of integrity of the plasma membrane. Our laboratory does not use PVP for ICSI [21]. It also may be reducing the ROS production.

Day 5 ROS levels in culture media were similar between embryos from IVF and ICSI procedures. The G series culture media is a sequential set of embryo culture media designed to mimic in vivo conditions. Embryos were cultured in G1™ v5 medium on days 1–3 and in G2™ v5 medium on days 3–5 or 6. Because the culture medium is replaced on day 3, the potential cellular sources of ROS in IVF and ICSI cycles are minimized.

Although our study showed that ROS levels in media may not be an indicator for embryo selection in IVF/ICSI, however, non-invasive embryo selection such as various biophysical, biochemical, and physicochemical markers of embryo development have been identified. These markers differ significantly between embryos that result in implantation and pregnancy and those that do not [4, 22]. Further studies are needed to determine whether ROS levels in culture media, which are indicative of several of these markers, play a role in embryo selection.

In conclusion, the utmost care should be exercised to avoid inducing excessive ROS production in ART laboratory settings. However, ROS levels in culture media may not be an effective indicator of embryo selection for IVF.
